# The JAK1/JAK2 inhibitor ruxolitinib inhibits mediator release from human basophils and mast cells

**DOI:** 10.3389/fimmu.2024.1443704

**Published:** 2024-08-12

**Authors:** Remo Poto, Leonardo Cristinziano, Gjada Criscuolo, Caterina Strisciuglio, Francesco Palestra, Gianluca Lagnese, Antonio Di Salvatore, Gianni Marone, Giuseppe Spadaro, Stefania Loffredo, Gilda Varricchi

**Affiliations:** ^1^ Department of Translational Medical Sciences, University of Naples Federico II, Naples, Italy; ^2^ Department of Oncology and Molecular Medicine, Istituto Superiore di Sanità (ISS), Rome, Italy; ^3^ World Allergy Organization (WAO) Center of Excellence, Naples, Italy; ^4^ Center for Basic and Clinical Immunology Research (CISI), University of Naples Federico II, Naples, Italy; ^5^ Department of Woman, Child and General and Specialistic Surgery, University of Campania “Luigi Vanvitelli”, Naples, Italy; ^6^ Institute of Experimental Endocrinology and Oncology (IEOS), National Research Council, Naples, Italy

**Keywords:** asthma, basophil, histamine, IL-4, IL-13, mast cell, polycythemia vera, ruxolitinib

## Abstract

**Introduction:**

The Janus kinase (JAK) family includes four cytoplasmic tyrosine kinases (JAK1, JAK2, JAK3, and TYK2) constitutively bound to several cytokine receptors. JAKs phosphorylate downstream signal transducers and activators of transcription (STAT). JAK-STAT5 pathways play a critical role in basophil and mast cell activation. Previous studies have demonstrated that inhibitors of JAK-STAT pathway blocked the activation of mast cells and basophils.

**Methods:**

In this study, we investigated the *in vitro* effects of ruxolitinib, a JAK1/2 inhibitor, on IgE- and IL-3-mediated release of mediators from human basophils, as well as substance P-induced mediator release from skin mast cells (HSMCs).

**Results:**

Ruxolitinib concentration-dependently inhibited IgE-mediated release of preformed (histamine) and *de novo* synthesized mediators (leukotriene C_4_) from human basophils. Ruxolitinib also inhibited anti-IgE- and IL-3-mediated cytokine (IL-4 and IL-13) release from basophils, as well as the secretion of preformed mediators (histamine, tryptase, and chymase) from substance P-activated HSMCs.

**Discussion:**

These results indicate that ruxolitinib, inhibiting the release of several mediators from human basophils and mast cells, is a potential candidate for the treatment of inflammatory disorders.

## 1 Introduction

The Janus kinase (JAK) family includes four cytoplasmic tyrosine kinases: JAK1, JAK2, JAK3, and tyrosine-protein kinase 2 (TYK2) (1, 2). JAKs are constitutively bound to several cytokine receptors and upon ligand binding to its receptor, JAKs phosphorylate downstream signal transducers and activators of transcription (STAT) (3, 4). The STAT family has seven members (STAT1, STAT2, STAT3, STAT4, STAT5A, STAT5B, and STAT6) (5), which have a major role in the regulation of hematopoietic and immune cells (2). The tyrosine kinase domain of JAKs is the site of catalytic activity and is blocked by first- and second-generation JAK inhibitors (6).

The JAK2-STAT5 signaling pathway is crucial for the activation growth and survival of mast cells (7, 8) and basophils (9–12). STAT5 also plays a role in IgE-mediated mast cell degranulation, making the JAK2-STAT5 pathway an appealing target for the inhibition of mast cell activation (8). Ruxolitinib, a JAK1/JAK2 inhibitor (13), has shown clinical benefits in polycythemia vera (PV) patients which carry an activity mutation of *JAK2* gene (i.e., V617F) (14–16). Ruxolitinib inhibits anaphylaxis in mice, and two studies reported a decrease in mast cell mediator-related symptoms in patients with systemic mastocytosis treated with ruxolitinib (17, 18). Several JAK1/2 and STAT5 inhibitors suppress the activation of mastocytoma cell lines (19, 20) and human basophils (21–23).

Human peripheral blood basophils share some similarities with tissue-resident mast cells (24, 25). Both cell types express high-affinity immunoglobulin (Ig)E receptors (FcϵRI), contain basophilic granules in the cytoplasm, and release histamine and other inflammatory mediators (24). Several studies have demonstrated the distinct roles of basophils in allergic inflammation in both mice (26, 27) and humans (28–30). Atopic dermatitis and chronic spontaneous urticaria are characterized by chronic pruritus (31) and basophils are involved in their pathobiology (32) and likely contribute to itch (33). Itching is also a common symptom in PV, affecting more than 50% of patients (34, 35). Pruritus can be an initial symptom or precede the development of hematologic manifestations (35–38). Importantly, Pieri and collaborators first demonstrated that basophils from JAK2 V617F PV patients overexpressed CD63, a marker of basophil activation (39), compared to controls when challenged with IL-3 plus fMLP (21). Moreover, the JAK2 inhibitor compound AZD1480 reduced CD63 expression in basophils of PV patients in response to IL-3 plus fMLP.

Mast cells are in close anatomical association with myelinated and unmyelinated neural structures and blood vessels (40), forming an important functional unit that maintains homeostasis and responds to insults (41–44). A critical aspect of this multicellular crosstalk includes the interaction between mast cells and sensory nerves (45). Sensory nerves express neuropeptides (e.g., substance P, VIP) and neurotransmitters that facilitate neural-immune communication, leading to mast cell mediator release which subsequently activates sensory neurons *via* different receptors (33). Mast cell density in the skin was increased in JAK2 V617F transgenic mice compared to controls (46).

Ruxolitinib inhibits the catalytic activity of wild-type JAK2 as well as mutant JAK2 (6). This drug was approved for the treatment of MF by the US Food and Drug Administration (FDA) in 2011 and by the European Medicines Agency (EMA) in 2012, followed by the approval for the treatment of hydroxyurea-resistant or -intolerant PV in 2014. Recent evidence demonstrates that ruxolitinib inhibits the release of hexosaminidase and TNF-α from mast cell lines (47) and the expression on human basophils of CD300f induced by IL-3 (48). In this study, we have evaluated the *in vitro* effects of pharmacologic concentrations of ruxolitinib on IgE-mediated release of proinflammatory mediators (histamine and LTC_4_) and cytokines (IL-4 and IL-13) from highly purified human basophils. Additionally, we have examined the effects of ruxolitinib on IL-3-mediated release of cytokines (IL-4 and IL-13) from basophils and on substance P-induced secretion of several preformed mediators (histamine, tryptase, and chymase) from human skin mast cells.

## 2 Materials and methods

### 2.1 Reagents

Bovine serum albumin, human serum albumin, piperazine-N, N’-bis (2-ethanesulfonic acid) (Pipes), hyaluronidase, chymopapain, elastase type I, substance P, LTC_4_ (Sigma Chemical Co., St. Louis, MO, USA), and ruxolitinib (Cambridge Bioscience, Cambridge, UK) were commercially obtained. Ruxolitinib was dissolved in ethanol at the concentration of 13 mg/ml. Collagenase (Worthington Biochemical Co., Freehold, NJ, USA), Hanks’ balanced salt solution and fetal calf serum (FCS), Iscove modified Dulbecco medium (IMDM) (GIBCO, Grand Island, NY, USA), human recombinant IL-3 (R & D System, Minneapolis, MN, USA), deoxyribonuclease I and pronase (Calbiochem, La Jolla, CA, USA), Percoll^®^ (Pharmacia Fine Chemicals, Uppsala, Sweden), HClO_4_ (Baker Chemical Co., Deventer, The Netherlands), (^3^H)-LTC_4_ (New England Nuclear, Boston, MA, USA) were commercially purchased. Basophil Isolation Kit II and CD117 MicroBead kit were obtained from Miltenyi, Biotec (Bologna, Italy). Anti-IgE produced by rabbit immunization with the Fc fragment of a human IgE myeloma (patient PS) and then absorbed with the IgE Fab (49) was a gift of Drs. Teruko and Kimishige Ishizaka (La Jolla Institute for Allergy and Immunology, La Jolla, CA, USA). Rabbit anti-LTC_4_ antibody was donated by Dr. Lawrence M. Lichtenstein (The Johns Hopkins University, Baltimore, MD, USA). Tryptase fluoroenzyme immunoassay (Phadia Diagnostic AB, Uppsala, Sweden) was kindly donated by Kabi Pharmacia (Milan, Italy).

### 2.2 Buffers

The Pipes buffer was made by 25 mM Pipes, 110 mM NaCl, 5 mM KCl, pH 7.4 and referred to as P buffer. P2CG contains, in addition to P buffer, 2 mM CaCl_2_ and 1 g/l dextrose (32) and was used for short-term (45 min) incubations of basophils and skin mast cells. PGMD contains 1 mM MgCl_2_, 10 mg/l DNase, and 1 g/l gelatin in addition to P buffer, pH 7.37 and was used to wash skin mast cells during the isolation. IMDM was used for long-term incubation of human basophils (4 hours for IL-4 and 16 hours for IL-13).

### 2.3 Purification and activation of human basophils

The study was approved by the Ethics Committee of the University of Naples Federico II (198/18), and written informed consent was obtained from all subjects involved in the study according to the recommendations from the Declaration of Helsinki. Basophils were isolated from peripheral blood of healthy volunteers (26% females), aged 19-44 years, undergoing hemapheresis at the University of Naples Federico II. Buffy coats were subjected to double-Percoll density centrifugation, which produced basophil-depleted cell and basophil-enriched cell suspensions (50, 51). Basophils were purified from the basophil-enriched cell suspensions using the Basophil Isolation Kit II (Miltenyi, Biotec, Bologna, Italy). Duplicate basophil aliquots, with a purity of ≥ 98% assessed by Alcian blue staining (52) were resuspended in P2CG and the cell suspension were placed in 12 x 75 mm polyethylene tubes and warmed to 37°C; anti-IgE (10^-1^ μg/ml) was added, and incubation was continued for 45 min at 37°C (53). At the end of incubations, cells were centrifuged (1000 g, 22°C, 2 min) and the supernatants were stored at -20°C for subsequent assay of histamine and LTC_4_ (54). Histamine was expressed as percent of the total content assessed in samples lysed with the addition of 2% HClO_4_, minus the spontaneous release (53, 55). LTC_4_ was analyzed by radioimmunoassay. Individual histamine and LTC_4_ release values were the means of duplicate determinations, replicates differing from each other by < 5%. In experiments evaluating the release of cytokines, basophils with purity ≥ 99% were incubated at 37°C for 4 hours (IL-4) or 16 hours (IL-13) (56) in IMDM in the presence of anti-IgE (10^-1^ μg/ml) or IL-3 (10 ng/ml). At the end of incubations, the cell-free supernatants were harvested and stored at -20°C for subsequent assay of IL-4 and IL-13 by ELISA (56).

### 2.4 Purification and activation of human skin mast cells

Skin samples were obtained from female patients, aged 20-58 years, undergoing either elective cosmetic surgery or mastectomy for breast cancer (54). The subcutaneous fat was eliminated by blunt dissection and skin tissue was cut into 1-2 mm fragments and dispersed into single cell suspension as previously described (54). Yields with this technique ranged between 0.1 and 0.8 x 10^6^ skin mast cells/g of wet tissue. At the end of this procedure, skin mast cell (HSMC) purities were between 4% and 8%. HSMCs were purified using a CD117 MicroBead Kit cell sorting system (Miltenyi Biotech, Bologna, Italy) according to the manufacturer’s instructions, reaching purities between 91% and 96% (54). Duplicate aliquots of purified HSMCs were suspended in P2CG and 0.3 ml of the cell suspensions were placed in 12 x 75 mm polyethylene tubes at 30°C; 0.2 ml of each prewarmed stimulus (substance P) was added, and incubation was continued at 30°C for 45 min (57). Mediator release from HSMCs is optimal at 30°C (54, 58). At the end of incubations, cells were centrifuged (1000 g, 22°C, 2 min) and the supernatants were stored at -20°C for subsequent assay of histamine, tryptase, and chymase.

### 2.5 Assay of histamine and LTC_4_

Histamine concentrations in supernatants of basophils and HSMCs were measured in duplicate samples with an automated fluorometric technique (32, 59). LTC_4_ was assayed in duplicate samples as previously described (60). The anti-LTC_4_ antibody is highly specific, with less than 1% cross-reactivity to other eicosanoids (60, 61). All determinations were run from duplicate samples against a standard curve also in duplicate. In calculating net LTC_4_ release, spontaneous release of LTC_4_ from basophils was always subtracted.

### 2.6 Assay of tryptase and chymase

Tryptase concentrations were measured in duplicate samples by fluoroenzyme immunoassay (FEIA) using Uni-CAP100 (Phadia Diagnostics AB, Uppsala, Sweden) as previously described (62). Chymase concentrations in supernatants of HSMCs were measured by DuoSet™ ELISA (R&D Systems, Minneapolis, MN, USA). The ELISA detection range was 100-8,000 pg/ml).

### 2.7 Assay of IL-4 and IL-13

IL-4 and IL-13 concentrations were assessed in duplicate samples using ELISA kits according to manifacturer’s instructions (Quantikine Elisa Kit) (R & D Systems, Minneapolis, MN, USA). The ELISA detection range was 31-2,000 pg/ml (IL-4) and 125-4,000 pg/ml (IL-13).

### 2.8 Assay of lactate hydrogenase

Lactate hydrogenase (LDH) concentrations were assessed in duplicate samples using LDH activity assay kit according to manufacturer instructions (Thermo Fischer Scientific, Monza, Italy).

### 2.9 Statistical analysis

Data were analyzed with the GraphPad Prism 9 software package (GraphPad Software, La Jolla, CA, USA). Values are expressed as mean ± SD (standard deviation of the mean). Normality tests (Shapiro-Wilk and Kolmogorov-Smirnov tests) were performed through GraphPad Prism 9 software. Since the normal distribution of the results was demonstrated, we performed one-way analysis of variance (ANOVA) (63). Correlations between two variables were assessed by Spearman’s rank correlation analysis and reported as coefficient of correlation (r). Values of *p* ≤ 0.05 were considered significant. A log concentration-inhibition curve for mediator release (histamine, LTC_4_, IL-4, IL-13, tryptase, and chymase) was constructed by plotting the log concentration of ruxolitinib against percent inhibition of release. IC_50_ values were assessed by interpolation.

## 3 Results

### 3.1 Effects of ruxolitinib on IgE-mediated release of mediators from human basophils

**Figure 1:**
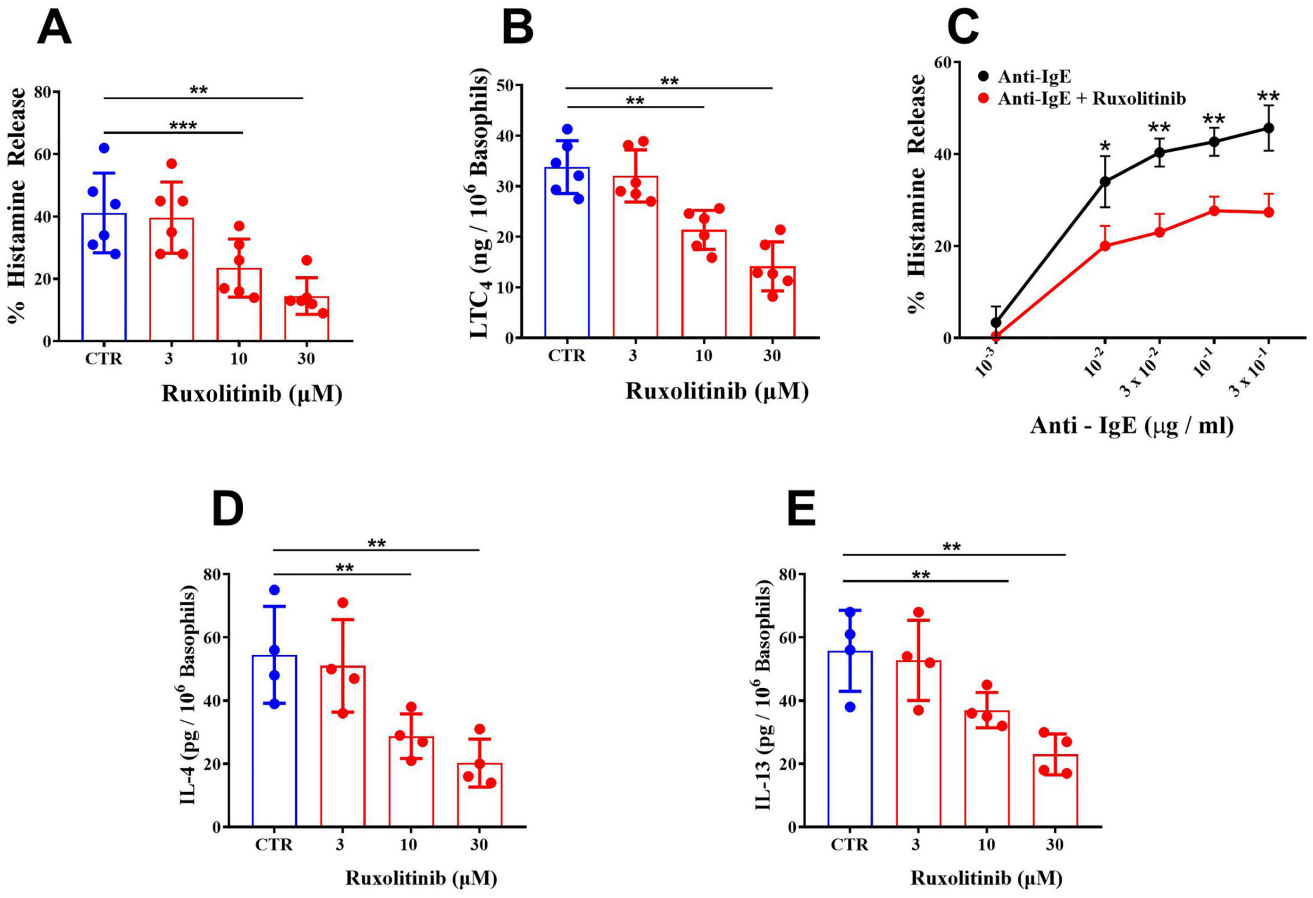
**(A)** Effects of increasing concentrations of ruxolitinib on anti-IgE-mediated histamine release from human basophils. Cells were preincubated (30 min, 37°C) with or without the indicated concentrations of ruxolitinib and then challenged (45 min, 37°C) with anti-IgE (10^-1^ μg/ml). Each bar represents the mean ± SD of six experiments with different preparations of basophils. **p < 0.01; ***p < 0.001 compared with histamine release in the absence of ruxolitinib (CTR). **(B)** Effects of increasing concentrations of ruxolitinib on anti-IgE-mediated LTC_4_ release from human basophils. Cells were preincubated (30 min, 37°C) with or without the indicated concentrations of ruxolitinib and then challenged (45 min, 37°C) with anti-IgE (10^-1^ μg/ml). Each bar represents the mean ± SD from six experiments with different preparations of basophils. **p < 0.01; compared with histamine release in the absence of ruxolitinib (CTR); **(C)** Effects of increasing concentrations of anti-IgE, alone or preincubated (30 min, 37°C) with ruxolitinib (10 μΜ) on histamine release from basophils. Cells were preincubated (30 min, 37°C) with or without ruxolitinib (10 μΜ) and then challenged (45 min, 37°C) with increasing concentrations of anti-IgE (10^-3^ – 3 x 10^-1^ μg/ml). Each point represents the mean ± SD from three experiments from different preparations of basophils. *p < 0.05; **p < 0.01. Effects of increasing concentrations of ruxolitinib on anti-IgE-mediated IL-4 **(D)** and IL-13 **(E)** release from human basophils. Cells were incubated with or without (CTR) the indicated concentrations of ruxolitinib and then challenged (4 hours for IL-4 and 16 hours for IL-13) with anti-IgE (10^-1^ μg/ml). Each bar represents the mean ± SD from four experiments with different preparations of basophils. **p < 0.01 compared with IL-4/IL-13 release in the absence of ruxolitinib (CTR).

In a first series of experiments, we evaluated the effects of ruxolitinib on IgE-mediated release of preformed (histamine) and *de novo* synthesized mediators (leukotriene C_4_: LTC_4_) from basophils purified from healthy donors. Basophils were preincubated (30 min, 37°C) with increasing concentrations of ruxolitinib (3 - 30 μM) and then challenged with an optimal concentration of anti-IgE (10^-1^ μg/ml). The concentrations of ruxolitinib used in these experiments reflect those achieved *in vivo* during treatment (64, 65) and are known to inhibit JAK1/JAK2 in human blood cells (13). These ruxolitinib concentrations did not affect the spontaneous release of LDH and histamine from basophils. Moreover, the vehicle (ethanol) corresponding to the highest concentrations of ruxolitinib (30 μM) did not affect the spontaneous or anti-IgE-mediated release of mediators (LDH, histamine, and IL-13) from basophils (data not shown). Ruxolitinib caused a concentration-dependent inhibition of histamine release from basophils activated by anti-IgE (**Figure 1A**). The inhibition ranged from approximately 4% at 3 μM to 80% at 30 μM, with an IC_50_ of 13.60 ± 3.93 μM.

IgE-mediated activation of basophils induces the *de novo* synthesis of LTC_4_ (66), a proinflammatory and vasoactive mediator implicated in several inflammatory disorders (67, 68) and angiogenesis (60, 69). The pharmacologic modulation of *de novo* synthesized mediators from basophils and mast cells does not always parallel that of preformed mediators (e.g., histamine). **Figure 1B** shows that in the same experiments illustrated in **Figure 1A**, ruxolitinib (3 - 30 μM) induced a concentration-dependent inhibition (5 to 58%) of LTC_4_ release from anti-IgE-activated basophils. In these experiments, the IC_50_ for the inhibition of LTC_4_ release from basophils was 21.70 ± 6.73 μM.

We also evaluated the effects of ruxolitinib on histamine release induced by suboptimal (10^-3^ to 3 x 10^-2^ μg/ml) and supraoptimal concentrations of anti-IgE (3 x 10^-1^ μg/ml). **Figure 1C** shows that increasing the concentrations of anti-IgE (10^-3^ to 3 x 10^-1^ μg/ml) induced a progressive increase in the percentage of histamine release from basophils. When basophils were preincubated (30 min, 37°C) with a suboptimal concentration (10 μM) of ruxolitinib, there was a significant inhibition of histamine release from basophils activated by all tested concentrations of anti-IgE.

### 3.2 Effects of ruxolitinib on IgE-mediated release of cytokines from human basophils

IgE-mediated activation of basophils results in the release of Type (T)-2 cytokines (IL-4 and IL-13) (70–73). The release of IL-4 from basophils is optimal after 4 hours of incubations, whereas IL-13 release is optimal after 16-18 hours of incubation (56, 71). To evaluate the effect of ruxolitinib on anti-IgE-induced IL-4 release, experiments were performed using purified (> 90%) basophils from healthy donors. As shown in **Figure 1D**, ruxolitinib (3 - 30 μM) caused a concentration-dependent inhibition of IL-4 release from basophils incubated (4 hours) with anti-IgE. The inhibition ranged from approximately 7% at 3 μΜ to 71% at 30 μΜ, with an IC_50_ of 13.20 ± 2.58 μΜ.

In parallel experiments, we evaluated the effects of graded concentrations of ruxolitinib (3 - 30 μM) on IL-13 release from anti-IgE-activated human basophils. Based on previous findings (56, 71), basophils were preincubated with ruxolitinib (30 min, 37°C) and then incubated for 16 hours at 37°C. **Figure 1E** shows that ruxolitinib concentration-dependently inhibited IL-13 release from anti-IgE-activated basophils. The inhibition ranged from 5% at 3 μΜ to approximately 59% at 30 μΜ, with an IC_50_ 21.60 ± 4.47 μΜ.

### 3.3 Effects of ruxolitinib on IL-3-induced cytokine release from human basophils

**Figure 2:**
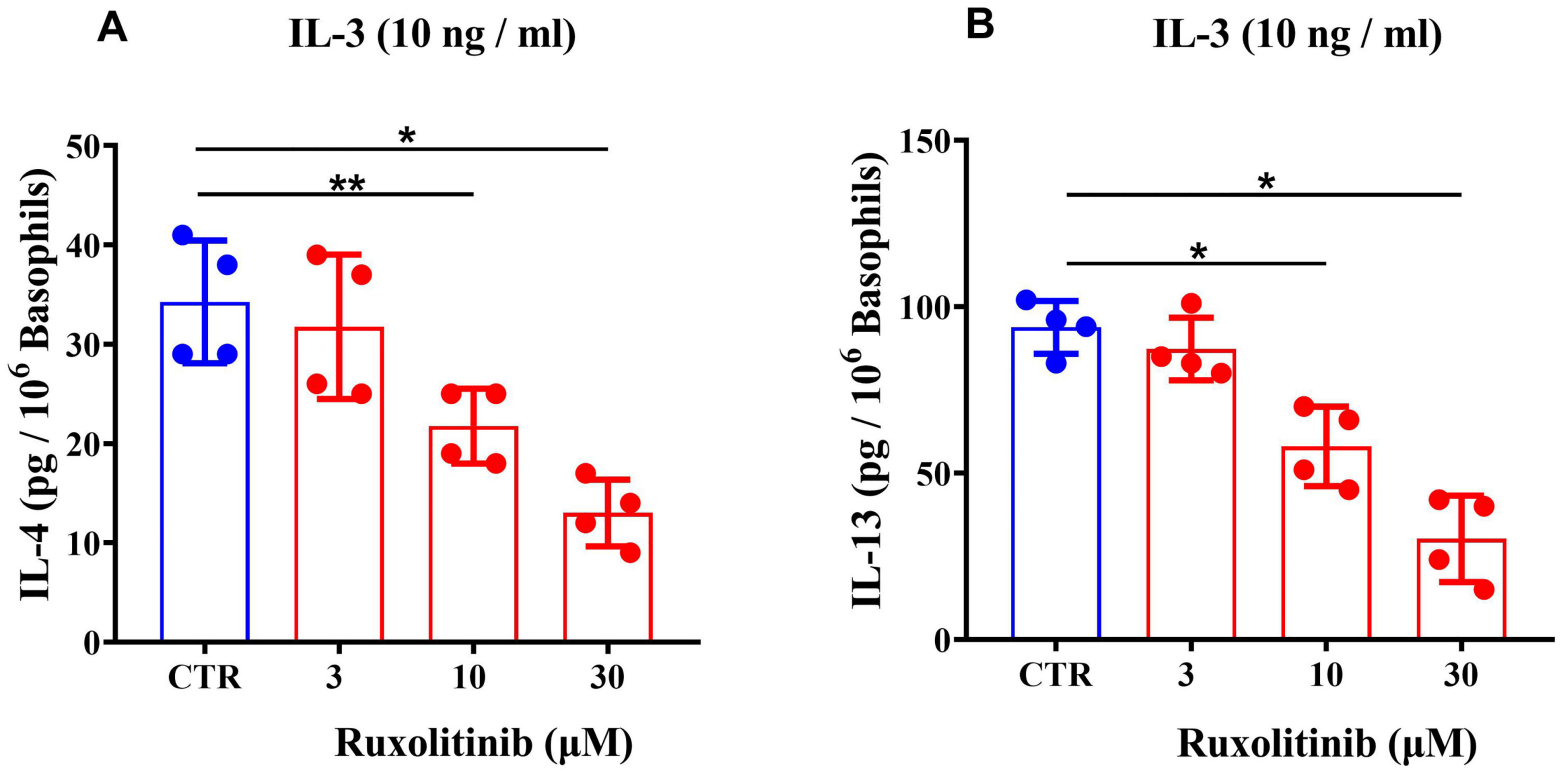
Effects of increasing concentrations of ruxolitinib on IL-3-mediated release of IL-4 **(A)** and IL-13 **(B)** from human basophils. Cells were incubated with the indicated concentrations of ruxolitinib and then challenged (4 hours for IL-4 and 16 hours for IL-13) with IL-3 (10 ng/ml). Each bar represents the mean ± SD from four different preparations of basophils. *p < 0.05; **p < 0.01 compared with IL-4/IL-13 release in the absence of ruxolitinib (CTR).

IL-3 induces the release of T2 high cytokines (IL-4 and IL-13) from basophils (10, 50, 56, 71, 74, 75) through the activation of the IL-3 receptor (76). We evaluated the effects of increasing concentrations (3 - 30 μM) of ruxolitinib on the release of IL-4 and IL-13 from basophils challenged with IL-3 (10 ng/ml). **Figure 2A** shows that ruxolinitib caused a concentration-dependent inhibition of IL-4 from IL-3-activated basophils. The inhibition ranged from approximately 8% at 3 μΜ to 61% at 30 μM, with an IC_50_ of 21.03 ± 5.55. The inhibition of IL-3-induced IL-13 release from basophils caused by ruxolitinib varied from 4% at 3 μΜ to 67% at 30 μΜ, with an IC_50_ of 18.60 ± 8.86 μΜ (**Figure 2B**).

### 3.4 Effects of ruxolitinib on substance P- mediated release of mediators from human skin mast cells

Mast cells are widely distributed in almost all human tissues (40, 53). The secretory granules of mast cells contain performed mediators, including histamine, tryptase and chymase (77, 78). Mast cells containing tryptase and chymase (MC_TC_) are predominant in human skin (HSMCs) (77, 78) and can be activated by substance P through the engagement of MAS-related G protein-coupled receptor-X2 (MRGPRX2) receptor (79). Substance P, a neuropeptide (80) which induces only the release of preformed mediators from HSMCs (54), is a potent endogenous pruritogen in mice and humans (81, 82).

**Figure 3:**
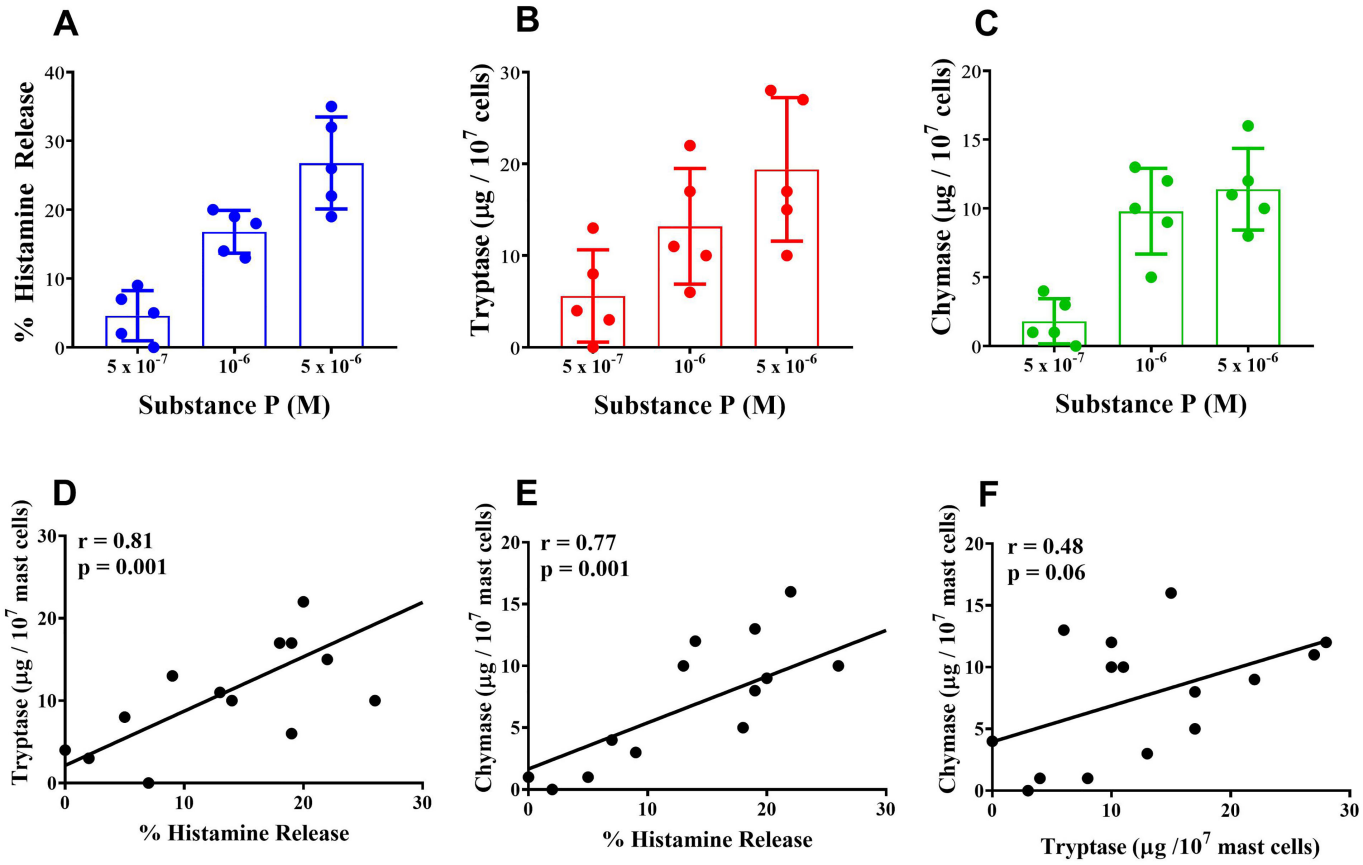
Effects of increasing concentration of substance P on the release of histamine **(A)**, tryptase **(B)** and chymase **(C)** from human skin mast cells (HSMCs). Cells were incubated (30 min, 30°C) with the indicated concentrations of substance P. Each bar represents the mean ± SD from five experiments with different preparations of HSMCs. Correlation between the release of histamine and tryptase **(D)**, histamine and chymase **(E)**, and tryptase and chymase **(F)** induced by the individual concentrations of substance P used in the five experiments.

In a series of five experiments, we evaluated the parallel release of histamine, tryptase and chymase from highly purified (> 90%) HSMCs challenged *in vitro* with increasing concentrations of substance P. Substance P (5 x 10^-7^ – 5 x 10^-6^ M) induced the concentration-dependent release of histamine (**Figure 3A**), tryptase (**Figure 3B**), and chymase (**Figure 3C**) from HSMCs. There was a linear correlation (r = 0.81; *p* < 0.001) between the release of histamine and tryptase from substance P-activated HSMCs (**Figure 3D**). Similarly, there was a linear correlation (r = 0.77; *p* < 0.001) between histamine and chymase release from HSMCs (**Figure 3E**). No significant correlation (r = 0.48; NS) was found between tryptase and chymase release from HSMCs induced by substance P (**Figure 3F**).

**Figure 4:**
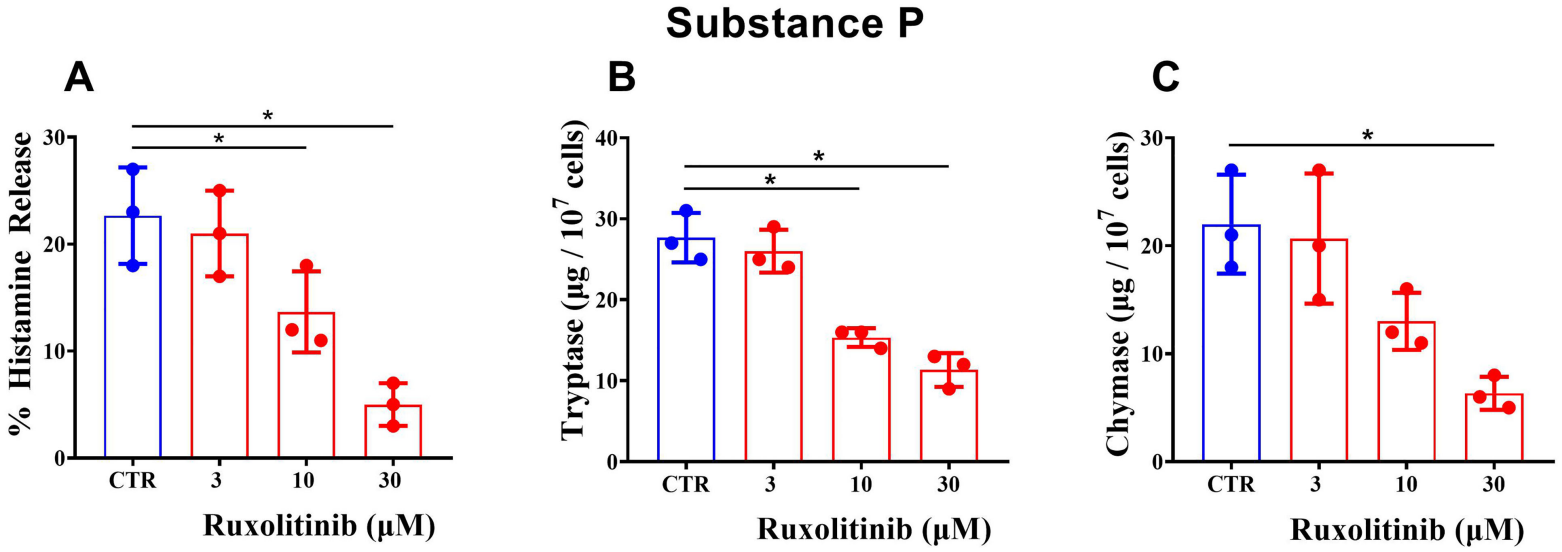
Effects of increasing concentrations of ruxolitinib on substance P-mediated release of histamine **(A)**, tryptase **(B)**, and chymase **(C)** from human skin mast cells (HSMCs). Cells were preincubated (30 min, 30°C) with the indicated concentrations of ruxolitinib and then challenged with buffer alone (CTR) or with substance P (5 x 10^-6^ M) (30 min, 30°C). Each bar represents the mean ± SD from three experiments with different preparations of HSMCs. *p < 0.05 compared with histamine/tryptase/chymase release in the absence of ruxolitinib (CTR).

In a next group of experiments, we compared the effects of increasing concentrations of ruxolitinib (3 - 30 μΜ) on the release of histamine, tryptase, and chymase from purified HSMCs activated by substance P (5 x 10^-6^ M). **Figure 4A** shows that ruxolitinib (3 - 30 μΜ) caused a concentration-dependent inhibition of histamine release from substance P-activated HSMCs. Similarly, in the same experiments, ruxolitinib inhibited the release of both tryptase (**Figure 4B**) and chymase (**Figure 4C**) from substance P-activated HSMCs. The IC_50_ for histamine (13.5 ± 2.29 μΜ), tryptase (17.7 ± 6.82 μΜ), and chymase (13.87 ± 2.60 μΜ) did not differ significantly.

## 4 Discussion

This study demonstrates that ruxolitinib inhibits the IgE-mediated release of preformed (histamine) and *de novo* synthesized proinflammatory mediators (LTC_4_) from highly purified human basophils. Furthermore, ruxolitinib inhibits the IgE- and IL-3-mediated release of cytokines (IL-4 and IL-13) from human basophils. Finally, ruxolitinib inhibits the release of several preformed mediators (histamine, tryptase, and chymase) from HSMCs activated by substance P.

Pharmacologic concentrations of ruxolitinib (64, 65), known to inhibit JAK1/2 in human blood cells (13), inhibited the release of histamine and cytokines induced by IgE cross-linking and IL-3, which activate distinct membrane receptors on basophils. Anti-IgE cross-links IgE bound to FcϵRI (83) and the JAK2-STAT5 signaling pathways play a critical role in IgE-mediated activation of basophils (9–12). IL-3 activates the heterodimeric receptor comprising the βc receptor and a cytokine-specific α chain (IL-3Rα) (76). The βc chain is the primary signaling component of the IL-3 receptor, while the specificity of IL-3 is determined by IL-3Rα. The cytoplasmic tail of βc chain binds mainly to JAK2, which phosphorylates and activates STAT5 (76). While JAK2 plays a central role in phosphorylating the βc (84, 85), JAK1 is also involved in mediating some βc chain signaling (86, 87). Collectively, these findings explain the inhibitory effects of ruxolitinib, a JAK1/2 inhibitor ^6^ on the anti-IgE- and IL-3-mediated release of cytokines from basophils.

Hermans and collaborators demonstrated that ruxolitinib inhibited the release of β-hexosaminidase from the human mast cell line LAD2 activated by substance P (47). Moreover, they found that ruxolitinib inhibited the release of TNF-α induced by the Ca^2+^ ionophore A23187 and MCP-1 production caused by substance P from the mast cell line HMC-1. We have extended their findings showing that ruxolitinib inhibited the IgE- and substance P-induced release of mediators from human basophils and HSMCs, respectively. These findings may have translational relevance in different inflammatory disorders in which basophils, mast cells, and their mediators play a pathogenic role.

Ruxolitinib is effective for the treatment of PV (88), a myeloproliferative neoplasm frequently associated with refractory and severe pruritus (89). Histamine and tryptase released from basophils and skin mast cells are involved in the pathophysiology of pruritus in atopic dermatitis (90, 91). Consistent with our findings, ruxolitinib is emerging as an effective therapy for the treatment of pruritus not only for patients with PV but also in human and experimental dermatitis (92).

It is known that *de novo* synthesized (LTC_4_) and preformed (histamine, tryptase, chymase) proinflammatory mediators play a role in skin inflammatory disorders (93). Moreover, T2-high cytokines, IL-4 and IL-13, play a key role in the pathophysiology of skin inflammation (94), such as atopic dermatitis. It has been recently demonstrated that ruxolitinib cream is effective in the treatment of adults and adolescents with atopic dermatitis (95–97). Activation of both resident skin mast cells and infiltrating basophils plays a key role in atopic dermatitis pathobiology (98, 99). In this study, we found that ruxolitinib inhibits the release of several preformed mediators such as histamine, tryptase, and chymase from substance P-activated HSMCs. There was a linear correlation between the release of histamine and both tryptase and chymase from HSMCs activated by substance P. These results are consistent with the notion that these preformed mediators are stored in cytoplasmatic compartments of HSMCs (100). Our findings showing an inhibitory effect of ruxolitinib on the release of proinflammatory mediators and T2-high cytokines from basophils and mast cells may explain, at least in part, the efficacy of this drug in the treatment of atopic dermatitis (95–97).

LTC_4_ and histamine are involved in lung inflammatory disorders (101). Furthermore, IL-4 and IL-13 play a critical role in asthma pathobiology (102). Recent evidences indicate that ruxolitinib reduces airway inflammation and airway hyperresponsiveness in different murine models of asthma (103, 104). The inhibitory effects of ruxolitinib on the *in vitro* release of histamine, LTC_4_, and T2-high cytokines (IL-4 and IL-13) from human basophils suggest that future studies should investigate the safety and efficacy of systemic or topical ruxolitinib in the treatment of the upper and lower airway inflammation.

Several studies have recently demonstrated that ruxolitinib inhibits *in vitro* and *in vivo* the release of different cytokines and chemokines from immune and structural cells involved in airway inflammation. In particular, ruxolitinib inhibits the release of IL-6 from human fibroblasts *in vivo* (105) and the production of IL-6, TNF-α and CXCL8 from monocyte-derived macrophages (MDM) *in vitro* (106–108), as well as IL-6 and TNF-α from human lung macrophages (109) and LAD2 cells (47). Ruxolitinib also inhibits the release of CCL5, a chemokine involved in asthma exacerbations, from bronchial epithelial cells *in vitro* (110). Our results extend previous findings showing for the first time that pharmacologic concentrations of ruxolitinib inhibit the release of T2 cytokines (IL-4 and IL-13) from human basophils.

Systemic mastocytosis is a rare clonal myeloproliferative neoplasm characterized by the proliferation and activation of mast cells (62, 111). Mast cell activation leads to the release of cytokines, histamine, and tryptase causing pruritus, flushing, hypotension and even shock (62, 111). Preliminary findings reported that ruxolitinib improved symptoms and quality of life in patients with systemic mastocytosis (17, 18). Our findings indicating that ruxolitinib inhibits mediator release from skin mast cells suggest that the potential properties of this drug require further exploration in mastocytosis.

Ruxolitinib has been approved by FDA and EMA for the treatment of myelofibrosis in patients with PV. Several preclinical studies have demonstrated the efficacy of systemic or topical JAK inhibitors in different animal models of lung inflammation (112). The modulation of a wide spectrum of inflammatory and immunomodulatory cytokines released by human mast cells, basophils, macrophages, and fibroblasts by ruxolitinib suggests that this drug is a potential candidate for the treatment of several inflammatory diseases beyond PV.

## Data Availability

The raw data supporting the conclusions of this article will be made available by the authors, without undue reservation.
